# Measurement and determinants of financial protection in health in Afghanistan

**DOI:** 10.1186/s12913-021-06613-y

**Published:** 2021-07-04

**Authors:** Ilker Dastan, Asiyeh Abbasi, Chokri Arfa, Mir Najmuddin Hashimi, Said Mohammad Karim Alawi

**Affiliations:** 1 World Health Organization, Tajikistan Country Office, Dushanbe, Tajikistan; 2 Statistical Centre of Iran, Tehran, Iran; 3 National Institute of Labor and Social Studies, University of Carthage, Tunis, Tunisia; 4 World Health Organization, Afghanistan Country Office, Kabul, Afghanistan; 5 Ministry of Public Health (Afghanistan), Kabul, Afghanistan

**Keywords:** Health financial protection, Catastrophic health expenditure, COVID-19, Afghanistan, Poverty

## Abstract

**Background:**

Out of pocket (OOP) payments for health are significant health financing challenges in Afghanistan as it is a source of incurrence of catastrophic health expenditure (CHE) and impoverishment. Measuring and understanding the drivers and impacts of this financial health hardship is an economic and public health priority, particularly in the time of COVID-19. This is the first study that measures the financial hardship and determines associated factors in Afghanistan.

**Methods:**

Afghanistan Living Conditions Survey data for 2016–2017 was used for this study. We calculated incidence and intensity of catastrophic health expenditure by using different thresholds ranging from 5 to 40% of total and nonfood consumption and subsequent impoverishment due to OOPs. Logistic regression was used to assess the degree to which Afghan households are protected from the catastrophic household expenditure.

**Results:**

Results revealed that 32% of the population in Afghanistan incurred catastrophic health expenditure (as 10% of total consumption) and when healthcare payments are netted out of household consumption, the Afghan population live in extreme poverty ($1.9 in 2011 PPP), increased from 29 to 36%. Based on our findings from logistic regression in Afghanistan, having an educated head or being employed are protective factors from financial hardship while having a female head, an elderly member, a disabled, or a sick member are the risk factors of facing catastrophic health expenditure. Moreover, the people living in rural/nomadic areas or facing an economic shock are more likely to face catastrophic health expenditure and hence to be impoverished due to direct OOPs on health.

**Conclusions:**

The high rate of poverty and catastrophic health expenditure in Afghanistan emphasizes the need to strengthen the health financing system. Although Afghanistan has made great efforts to support households against health expenditure burden during the pandemic, households are at higher risk of poverty and financial hardship due to OOPs. Therefore, there is need for more financial and supportive response policies by providing a better and easier access to primary health services, extending to all entitlement to health services particularly in the public sector, eliminating user fees for COVID-19 health services and suspending fees for other essential health services, expanding coverage of income support, and strengthening the overall health financing system.

## Background

Protecting households against financial hardship is a key function of every health system and has been conceptualized as ensuring financial protection (FP), which has been defined by the World Health Organization WHO as the state wherein “direct payments to obtain health services should  not expose people to financial hardship and should  not threaten their living standards.” Ensuring FP is a key component of achieving Universal Health Coverage (UHC), which has received increased political commitment worldwide through the inclusion of a target to achieve UHC among the Sustainable Development Goals (SDGs 3.8). The goal of UHC is to ensure that every individual and community, regardless of their circumstances, should receive the health services they need without having financial hardship [1, 2]. Health financing does not only cover the existence of specific problems but the concern for an overall income/expenditure balance. It has become an approach to assess progress towards UHC. Issues of financial protection and equity are necessary for health financing strategy for all countries in the aim of reducing OOPs burden. It enables to identify and address the harmful consequences of fragmentation in health financing arrangements and ensures that these policy instruments are aligned with UHC goals [3].

Financial health protection has become an essential component in the strategy development of health financing in order to achieve progress towards UHC. The related indicators allow evaluating the burden of the high OOPs and the weak financial arrangements [3]. All economic and health shocks that could arise from diseases and pandemics could contribute to increase in financial hardship in low and middle-income countries such as Afghanistan, where OOP payments for health are very high coupled with ineffective health coverage. The situation may get worse in view of coronavirus outbreak that has generated loss of jobs, of revenue, and a decrease in the economic activities accompanied by an ineffective running health system. In Afghanistan, households are exposed to the financial protection that could be easily detected through the high direct contribution of households in the current health expenditures. In 2018, the share of OOP as a percent of current health expenditure is was estimated to be 78% of which is the second highest in the world [4]. Indisputably, there is a significant proportion of households that are impoverished due to the OOPs on health and this could be exacerbating during the current COVID-19 outbreak.

Even though Afghanistan has made progress in achieving UHC it is still among the countries with the highest level of OOPs and lowest public spending in health [5]. Evaluating the financial health protection through the well-known indicators of CHE and providing evidence on how to reduce health inequity in Afghanistan will certainly highlight what is required to manage the shocks of the COVID-19 crisis.

In most developing countries, protecting households from the financial burden of diseases is considered an effective means to progress towards UHC [6]”. Since 2016, the Government of the Islamic Republic of Afghanistan has been committed to achieving the SDGs and UHC target in health settings. Thus, the Ministry of Public Health as the main steward of the health system has introduced changes in the health financing system through the definition of the Basic Package of Health Services (BPHS) and Essential Package of Hospital Services (EPHS). Also, under the system Enhancement for Health Action in Transition Project, resources allocated for the BPHS and the EPHS (on-budget) are generated under one umbrella “Afghanistan Reconstruction Trust Fund platform”, which covers the totality of the country and allowing harmonization and implementation of benefit packag and payment arrangements across the provinces [7]. A Grant Contracting and Management Unit under the Ministry of Public Health contracts out healthcare services to nongovernmental organizations in 31 provinces while retaining responsibility for service delivery in the remaining three provinces through a contracting-in modality.

Health services are financed by three donors: The World Bank (WB), the European Union, and the United States Agency for International Development. In 2017, per capita current health expenditure stood at US$ 81, representing 12% of Gross domestic product (GDP), the highest rate in the Eastern Mediterranean Region of the World Health Organization. However, in 2017, roughly 75% of healthcare in Afghanistan is procured through OOP payments rather than through pooled/prepayment financing mechanisms, such as formal health insurance or government tax-funding . The share of OOP in health expenditure is significantly higher than the average of 40% in low and lower-middle-income countries. In 2017, 19.4% of total health expenditures and 58.3% of total government expenditure came from foreign financing [8]. During the period 2006-2017, average, household expenditure on medicines, diagnostics and aboard treatments were estimated respectively to be 54%, 35% and 10% of the total households spending on health. The share of domestic public expenditure in total public expenditure remained stable between 2013 and 2015 at an average of 2% – around US$ 3.1 per capita, with a clear fragmentation in raising revenue and pooling [9]. In Afghanistan, several health facilities –mainly hospitals– are directly supported through bilateral donors or managed by other government ministries such as Ministry of Defense, Ministry of Interior, and Ministry of Higher Education.

Regarding financial protection in health, very limited evidence is available in Afghanistan. A study on the care-seeking pattern using household survey 2004 shows high rates of reported care-seeking and a high level of OOPs. It also shows that 30% of households were reported to borrow money or sell assets/land to pay for healthcare services and these figures are almost twice for households in the poorest versus the rich quintile. The high OOPs are a consequence of the ineffective compulsory arrangements, and health protection in Afghanistan [10]. Under the BPHS, all Afghan citizens have the right to receive services at five standard types of health facilities. The EPHS establishes a standard service package for each hospital level; provides staffing guidelines for hospitals; promotes a referral system to integrate the BPHS facilities with hospitals and the essential medicines list. It is mandated that all hospitals providing the EPHS should have four clinical functions: medicine, surgery, pediatrics, and obstetrics and gynecology. Public health services are free of charge for all, according to the Afghan constitution and health law.

Gaps in service's coverage are also linked to a set  of barriers such as availability, affordability, and acceptability of services. The volatile security particularly in rural areas is a barrier. 57% of the population have access to health services within an hour’s walk. The household survey 2016–2017 showed that 86.7% of the population could access health services within 2 hours, through any type of transportation.

Since the beginning of March 2020, COVID-19 has spread rapidly all over Afghanistan. The WHO reported cases as of May 22, 2021; with more than 65,000 infections and 2700 deaths [11]. Afghanistan is struggling to create the required testing capacity being constrained by a lack of personnel and medical equipment. Invariably, the funding and equipment needs for the medical response is way off the ability of Afghanistan to cope [12]. The World Bank prediction indicates that poverty may increase from a baseline of 54.5% to up to 72% during COVID-19 [13]. The result of The Afghanistan Multidimensional Poverty Index based on Afghanistan Living Condition Survey (ALSC) (2016–2017) shows that half (51.7%) of Afghan population  are experiencing the multidimensional poverty. The rate of multidimensional poverty has a huge variation among region: 12% in Kabul and 81% in Baghdis. 83% of those live in rural areas. Nothing that 58% of those facing this poverty  in Afghanistan are aged under 18 years [14].

The purpose of this study is to evaluate the OOP effects on Afghanistan’s households using the Afghanistan Living Condition Survey (ALCS) 2016–2017. We measure ‘catastrophic health headcount’, ‘catastrophic payment gap’, and impoverishment due to healthcare payments using two international poverty lines (1.9$ and 3.2$ a day in 2011 PPP). Monitoring catastrophic health expenditures and impoverishment due to OOPs could provide aclear idea of FP. However, we need a deep analysis to determine the profile of  households that are facing highest risk and their coping mechanisms (borrowing, selling, etc.). Investigating these issues could help designing the interventions that will reduce financial hardship and inequity in the utilization of healthcare services, and will help identifying the ways to progress towards UHC. The multivariate logistic regression was used to determine the factors associated with the exposure to CHE. The remainder of the paper is organized as follows.

In next section, we introduce the method that shows households’ exposure to catastrophic health expenditure and impoverishment and the variables used to explore the determinants of CHE. In result Section, we provide the results and discuss them in the last section.

## Methods

The incidence of CHE is estimated as a share of total (or total nonfood) expenditure exceeding a threshold. Let *T*_*i*_ be OOP payments for health care, *x*_*i*_ be total household expenditure and *xnf*_*i*_ be household nonfood expenditure. Define an indicator, *E*_*i*_, which equals 1 if *T*_*i*_/*x*_*i*_ > *z*_*cat*_ and zero otherwise. Then an estimate of the headcount is given by where N is the sample size.

1 $$ {H}_{cat}=\frac{1}{N}{\sum}_{i=1}^N{E}_i $$

Another measure, the catastrophic payment overshoot, captures the average degree by which payments (as a proportion of total expenditure) exceed the threshold *z*_*cat*_. Define the household overshoot as *O*_*i*_ = *E*_*i*_((*T*_*i*_/*x*_*i*_) − *z*_*cat*_). Then the overshoot is simply the average:

2 $$ {G}_{cat}=\frac{1}{N}{\sum}_{i=1}^N{O}_i $$

Although *H*_*cat*_ captures only the incidence of any catastrophes occurring, *G*_*cat*_ additionally captures the intensity of the occurrence. They are related through the mean positive overshoot (*MPG*_*cat*_), which is defined as follows:

3 $$ {MPG}_{cat}=\frac{H_{cat}}{G_{cat}} $$

Lastly, the *MPG*_*cat*_ measures the intensity of CHE computed for the subsample of households with CHE in their quintile of equivalent expenditure [15]. Measures of catastrophic payments defined with respect to nonfood expenditure can easily be obtained by simply replacing *x*_*i*_ with *xnf*_*i*_ in the denominator of the OOP budget share. We examine the prevalence and intensity of CHE across equivalent household expenditure quintiles and other socio-economic characteristics.

The impoverishing health payment headcount (*H*_*pov*_) and gap (*G*_*pov*_) measures are obtained by comparing poverty estimates derived from household resources gross and net healthcare expenditures. Thus, the impoverishing payment headcount (*H*_*pov*_) represents the difference in the proportions of households below the poverty (or deep poverty) line before and after accounting for healthcare payments. The impoverishing gap (*G*_*pov*_) is the combined amounts by which poor households fail to reach the poverty line in the population, obtained by comparing intensity in poverty before and after paying for healthcare. Lastly, the two measures, *H*_*pov*_ and *G*_*pov*_ are related through the mean positive gap (*MPG*_*cat*_), which represents the intensity of impoverishment [16]. Because of the obvious sensitivity of impoverishing measures to the chosen poverty line, we use two alternative definitions; 3.2$ a day in 2011 PPP per person as a national poverty line, and 1.9$ a day in 2011 PPP per person as an international poverty line.

For further potential vulnerability, we explored the keys factors associated with CHE by employing the binary logistic regression. The logistic regression is used to test a hypothesis about the determinants or correlates of catastrophic due to OOP:

4 $$ 1\mathrm{n}\left(\frac{P(y)}{1-P(y)}\right)={\beta}_O+{\sum}_k{\beta}_k{X}_k $$

Where *P*(*y*) is the probability of household exposure to CHE, *β*_0_ is constant, *X* is a vector of *k* independents variables. The explanatory variables are a set of economic and demographic variables relating to the household or to the household head (Table 1 below) for which odds ratios were estimated. Note that all estimates were based on the sampling weights and were corrected for clustering.

**Table 1:** Statistical description of variables used (ALCS 2016-2017)

		Min	Mean	SE*	Max
Variables used to calculate CHE and Impoverishment					
OOP per capita per day		0.00	6.40	0.25	4965.7
Food expenditure per capita per day		0.14	22.97	0.14	986.3
Total Expenditure per capita per day		0.66	62.56	0.66	8033.9
Independent’s variable of the logistic regression					
Area of residence	Urban	0	24.93	0.31	1
	Rural	0	70.03	0.33	1
	Kuchi	0	5.05	0.16	1
Household head					
Household head Gender	Male	0	98.81	0.08	1
	Female	0	1.19	0.08	1
Household head Age	10-39 years	0	44.7	0.35	1
	40 plus	0	55.3	0.35	1
Household head Activity	Employed	0	61.33	0.35	1
	Unemployed	0	38.67	0.35	1
Household head completed Education	University	0	5.25	0.16	1
	Primary	0	22.32	0.3	1
	Illiterate	0	72.42	0.32	1
Number of household members	1 and 3	0	2.45	0.11	1
	4 and 6	0	36.21	0.34	1
	More than 6	0	61.34	0.35	1
Having an elderly person (60 years old and older)	≥ 1	0	14.18	0.25	1
	0	0	85.82	0.25	1
Having any child person (5 years old and younger)	≥ 1	0	71.01	0.32	1
	0	0	28.99	0.32	1
Having a member with disability	≥ 1	0	18.54	0.28	1
	0	0	81.46	0.28	1
Having OOP health expenditure	Poorest (Q1)	0	46.47	0.35	1
	Q2	0	58.22	0.35	1
	Median (Q3)	0	64.19	0.34	1
	Q4	0	67.1	0.33	1
	Richest	0	67.19	0.33	1
	Total	0	60.63	0.35	1

*Source:* Author calculations

*SE Standard Error

The data of the household’s survey (ALCS 2016–2017) was used. This survey generated evidence on population, poverty, food security, employment, housing, health, education, and a wide range of other issues. A total of 19,838 households and 155,680 individuals were sampled and used for this survey. Two-stage cluster design within each province: In total, 35 strata were identified, 34 for the provinces of Afghanistan and one for the nomadic (Kuchi) population.

The statistical description of the variables used is given in Table 1. The mean of OOP payment, food expenditure, and total expenditure are respectively 6.4 Afghani, 22.97 Afghani, 62.56 Afghani per capita per day in 2017. Table 1 presents the most commonly used variables in the literature [17] as drivers of CHE: area of residence, gender, age, employment and completed education, household size, a household with an elderly or children, household with OOP expenditure, and expenditure level. 70% of households were in rural areas, 1.2% of households were female-headed, 55% of household heads were above 40 years old, and 61% of household heads were employed. A large proportion of heads (72%) were illiterate. 61% of households had more than 6 members. 14% had at least one elderly person, 71% had at least one child and 19% had at least one person with a disability. The results also reveal that 61% have OOP health spending. This rate in the lowest quintiles (poorest households) is 46%, and in the upper quintiles (richest households) is 67%.

The survey provides a health proxy variable. The disability variable was derived from the response to the question “Does any member of the household have difficulty hearing, seeing, walking, with self-care, remembering, or communicating even using product aids”.

## Results

Table 2 presents the prevalence (headcounts) and intensity (overshoot) of CHE in Afghanistan for 2017, measured with 5 different threshold levels, ranging from 5 to 40%. Two models were considered, depending on whether CHE is measured through total expenditure or discretionary non-food expenditure. With total expenditure as a reference, in 2017, we found that as the intensity threshold level increases from 5 to 40%, the proportion of households experiencing CHE decreases sharply from 45.07 to 4.33%. According to the Mean Positive Gap, households are at risk of CHE, at a threshold of 25%, devote 36.54% (25% + 14.54%) of their total expenditure to health services while this proportion reaches 53.65% (40% + 13.65%) at the 40% threshold for the year 2017.

**Table 2:** Incidence and intensity of CHE, 2017

Threshold budget share of household total expenditure								
	5%	10%	15%	20%	25%	30%	35%	40%
Head count (H_cat_)	45.07	31.69	22.32	15.84	11.61	8.26	5.97	4.33
SE* (H_cat_)	0.48	0.45	0.40	0.38	0.33	0.28	0.24	0.21
Gap (G_cat_)	6.55	4.65	3.31	2.36	1.69	1.20	0.85	0.59
SE (G_cat_)	0.13	0.11	0.10	0.08	0.07	0.06	0.05	0.04
Mean positive gap (MPG)	14.54	14.67	14.83	14.92	14.54	14.49	14.16	13.65
SE (MPG)	0.22	0.26	0.30	0.35	0.39	0.43	0.47	0.51
Threshold budget share of household Nonfood expenditure								
	5%	10%	15%	20%	25%	30%	35%	40%
Head count (H_cat_)	51.64	43.99	37.17	30.69	25.25	20.45	16.59	13.53
SE (H_cat_)	0.47	0.48	0.48	0.47	0.44	0.42	0.39	0.35
Gap (G_cat_)	12.69	10.31	8.28	6.60	5.20	4.06	3.14	2.39
SE (G_cat_)	0.20	0.19	0.17	0.15	0.14	0.12	0.10	0.09
Mean positive gap (MPG)	24.58	23.43	22.28	21.49	20.60	19.86	18.92	17.65
SE (MPG)	0.30	0.31	0.33	0.34	0.35	0.35	0.36	0.39

*Source:* Author calculations

*SE Standard Error

The second specification assimilates the ability of households to pay for total non-food expenditures. This specification reveals the same results as those regarding total expenditure, at a relatively greater magnitude. It shows that 13.53% in 2017 of the sample consists of households experiencing CHE, at a threshold of 40%. At this threshold too, at-risk household allocation of total non-food expenditures to health care was 57.65 (40% + 17.65%) in 2017.

Table 3 presents the distribution of CHE based on demographic and socioeconomic factors. The rate of CHE is significantly higher among Kuchi, rural and large households as well as the households headed by a female, an unemployed or illiterate person, and households having at least one elderly or disabled member or a member with a disease. And also, the rate of CHE is higher among households with an economic shock (illness of working or other member or income shock). Those in the lowest expenditure quintile overshoot the 10% threshold of total expenditure, on average, 24.2% (10% + 14.2%) on health services compared to 28.6% (10% + 18.6%) for the highest quintile.

**Table 3:** Incidence of CHE by household’s characteristics, 2017

Variables		As 10% of total expenditure			As 40% of nonfood expenditure		
		H_cat_	GAP	MPG	H_cat_	GAP	MPG
Household head Gender	Male	31.6	4.6	14.6	13.4	2.4	17.6
	Female	36.3	6.3	17.4	21.9	4.3	19.9
Household head Age	10-39	31.9	4.6	14.5	13.4	2.3	17.5
	≥ 40	31.5	4.7	14.8	13.7	2.4	17.8
Household head Activity	Unemployed	33.0	5.3	16.0	15.7	3.0	18.8
	Employed	30.9	4.3	13.8	12.1	2.0	16.7
Household head completed Education	Illiterate	32.6	5.0	15.2	15.0	2.7	18.0
	Primary level	29.5	3.8	13.0	9.7	1.6	16.5
	High level	28.9	3.9	13.4	9.4	1.5	16.1
Area of residence	Urban	30.3	3.9	12.8	9.2	1.5	16.2
	Rural	31.3	4.7	15.1	14.2	2.5	17.6
	Kuchi	43.8	7.5	17.2	25.8	5.3	20.5
Having OOP health expenditure	Poorest	20.8	2.9	14.2	10.9	2.0	18.3
	Q2	30.1	4.0	13.4	14.1	2.2	15.7
	Median	34.7	4.5	13.1	13.9	2.1	15.3
	Q4	39.8	5.6	14.0	15.8	2.6	16.6
	Richest	33.1	6.1	18.6	13.0	3.0	23.0
Number of household members	1-2	24.2	5.3	21.9	15.6	3.3	20.8
	3-4	31.5	4.9	15.5	14.4	2.7	19.0
	≥5	32.1	4.5	13.9	12.9	2.1	16.6
Having an elderly member (60 years old and older)	0	30.7	4.5	14.5	13.0	2.3	17.4
	≥ 1	35.0	5.3	15.2	15.4	2.8	18.4
Having a member with disability	0	30.4	4.4	14.3	12.8	2.2	17.2
	≥ 1	37.2	5.9	15.9	17.0	3.3	19.3
An economic shock (illness of working or other member or income shock)	0	27.2	3.6	27.2	11.1	1.8	11.1
	≥ 1	43.9	7.3	43.9	20.4	3.9	20.4

*Source:* Author calculations

Similar patterns were observed when a comparison was held against the 40% of discretionary expenditure, of course, with more differences. It is apparent from the fraction of the households exceeding the threshold that CHE is significantly five times lower among the richest households than the poorest households.

The impoverishment effects of CHE are summarized in Table 4 according to World Bank’s poverty line and the total consumption basis. The results indicate that 29.39% of Afghan households live in extreme poverty (< 1.9$ a day in 2011 PPP). This percentage rises to 36.23% when healthcare payments are netted out of household consumption. Expressed as a percentage of the poverty line, the poverty gap increases from 2.7 of the 1.9$ (a day in 2011 PPP) line to 3.5 when healthcare payments are netted out of household consumption. Also, when healthcare payments are netted out of household consumption, the normalized mean positive poverty gap falls by 2.54%, indicating that the rise in the poverty gap is rather due to an increase in the number of households being brought into poverty, rather than a deepening of the poverty of the already poor. According to the other poverty line (3.2$ a day in 2011 PPP), 63.5% of households fell under the poverty line in 2017.

**Table 4:** Measures of impoverishing effects of healthcare payments, 2017

	Gross of healthcare payments (1)	Net of healthcare payments (2)	Differences	Relative [(3) / (1)] (%)
			Absolute	
			(3) = (2)-(1)	
Poverty line = $ 1.9 a day in 2011 PPP				
Poverty head count (H_pov_)-%	29.39 (0.539)*	36.23 (0.587)	6.84 (0.248)	23.28
Poverty gap (G_pov_)	2.68 (0.063)	3.52 (0.078)	0.84 (0.028)	31.25
Normalized poverty gap-%	8.13 (0.192)	10.67 (0.237)	2.54 (0.085)	31.25
MPG_pov_-%**	27.66 (0.332)	29.45 (0.357)	1.79	6.46
Poverty line = $ 3.2 a day in 2011 PPP				
Poverty head count (H_pov_) -%	63.49 (0.583)	70.03 (0.558)	6.53 (0.245)	10.29
Poverty gap (G_pov_)	13.47 (0.178)	15.97 (0.193)	2.5 (0.051)	18.54
Normalized poverty gap-%	24.25 (0.321)	28.74 (0.347)	4.5 (0.093)	18.54
MPG_pov_-%**	21.22 (0.164)	22.8 (0.166)	1.59	7.48

*Source:* Author calculations

*Standard errors are between parentheses

**Estimated by taking the mean gap over all households below the poverty line

Results of logistic regression of the determinants of CHE are presented in Table 5 for the two common thresholds, 10% of total expenditure, and 40% of discretionary expenditure for 2017. Based on the Chi-Square test (*p*-value<.0001), the model goodness-of-fit is satisfactory^1^, the odds ratio can be interpreted as the percentage increase of the CHE incidence compared to the reference group. The regression yielded a wide range of determinants linked with CHE. Households in rural and Kuchi (Nomadic) areas, households headed by a female, an unemployed or illiterate person, and households having at least one elderly or disabled member or a member with a disease especially circulatory system and endocrine diseases were more likely to experience CHE. Economic Shock in a household such as sickness of working member or other members, or losing the whole or a part of income increases the likelihood of CHE.

**Table 5:** Factors associated with catastrophic health expenditure in Afghanistan, 2017

	CHE as 10% of total expenditure				CHE as 40% of nonfood expenditure			
Parameter	Β	T	Pr > |t|	Odds Ratio	Β	T	Pr > |t|	Odds Ratio
Type of disease (reference = No member with diseases)								
Infectious and parasitic diseases	3.5	44.3	0.0	34.8	3.0	25.0	0.0	19.6
Circulatory system and endocrine disease	3.9	39.9	0.0	50.7	3.4	25.7	0.0	29.8
Respiratory system diseases	3.3	37.3	0.0	26.6	3.0	23.3	0.0	19.5
Other diseases	3.8	51.7	0.0	43.8	3.3	29.3	0.0	27.5
Household composition (reference = At least one member)								
Without elderly member	0.1	2.9	0.0	1.1	0.1	1.8	0.1	1.1
Without disable member	0.3	5.8	0.0	1.3	0.3	5.1	0.0	1.4
Economic Shock in household	0.8	17.6	0.0	2.1	0.8	14.4	0.0	1.3
Gender of household head (reference = Male)								
Female	0.5	2.9	0.0	1.7	0.7	3.1	0.0	1.9
Age of household head (reference =10-39 years old)								
>39 years old	-0.1	-3.1	0.0	0.9	-0.1	-1.0	0.3	1.0
Employment status (reference = Unemployed)								
Employed	-0.1	-3.3	0.0	0.9	-0.3	-4.8	0.0	0.8
Education level (reference = Illiterate)								
Primary level	-0.2	-3.6	0.0	0.8	-0.4	-5.1	0.0	0.7
High level	-0.3	-2.6	0.0	0.8	-0.4	-3.0	0.0	0.7
Area of residence (reference = Urban area)								
Rural	0.3	4.0	0.0	1.3	0.6	6.5	0.0	1.9
Kuchi (Nomadic)	0.8	6.6	0.0	2.3	1.4	8.8	0.0	3.9
Expenditure Quintile (reference = Q1 (Poorest Quintile))								
Q2	0.6	9.7	0.0	1.8	0.5	6.0	0.0	1.6
Q3	0.9	13.6	0.0	2.4	0.6	7.2	0.0	1.8
Q4	1.2	16.5	0.0	3.2	0.9	9.0	0.0	2.4
Q5 (Richest Quintile)	1.0	13.0	0.0	2.6	0.8	7.7	0.0	2.2
household size (reference =1-2 members)								
3-4 members	0.2	1.1	0.3	1.2	-0.2	-1.2	0.3	0.8
≥ 5	0.0	-0.3	0.8	1.0	-0.6	-3.0	0.0	0.6
Intercept	-1.7	-11.0	0.0	0.2	-2.4	-12.0	0.0	0.1
F value (goodness of fit test)	F: 26.48	DF: 16	Pr >0.000		F: 17.76	DF: 16	Pr >0.0000	

Results on the other thresholds are available upon request

An odds ratio equal to 1.65 for households with female head areas illustrates a 65% increase compared to households with male-headed households. Results also show a socioeconomic gradient in the probability of facing CHE: the odds decline to move from the upper to the lower expenditure quintiles, with households in the richest quintile being more likely to face CHE than those in the poorest quintile.

**Fig. 1:**
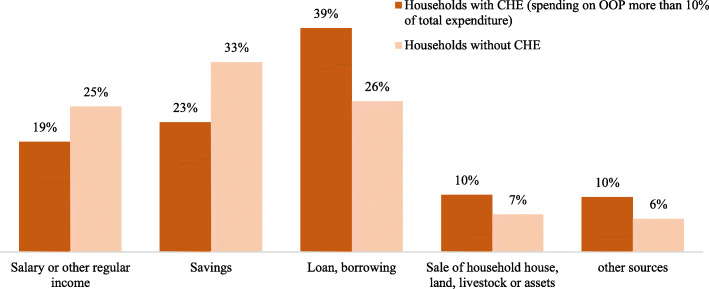
Source of payments for hospital service comparing households with/without CHE-2017

In ALCS, Afghan households are asked “What is the main source to pay for health care?” The strategies for households to cope with health costs can be listed as paying from regular income, sale of assets, savings, or borrowing money and taking loans. Our results show there is a different pattern between households with and without CHE in the source of payment for health care costs. In Afghanistan, households which have faced CHE employ different strategies. Figure 1 illustrates the source of payments for hospital expenditures comparing households with and without CHE. In Afghanistan, strategies of taking a loan (39%), using savings (23%), using regular salary (19%), and selling house, land, or assets (10%) were employed by households that experienced catastrophic health expenditures to cope with hospital costs. We can conclude that some of these strategies have caused households to fall deeper into poverty. A study in Cambodia showed that households that borrowed loans with high interest rates to treat their diseases remained indebted for a long period. Therefore, the catastrophic health expenditures not only bring about instantaneous shocks to the households, but they may also lead to further poverty as a result of the strategies used for coping with these expenses [18].

**Fig. 2:**
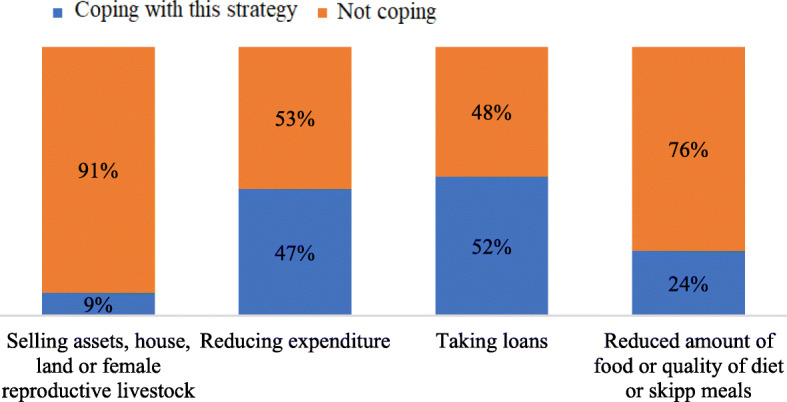
Different coping strategies against economic shocks (including illness of working member or other members or reduced income)

Generally, when faced with incidents that reduce income resources, as expected during the COVID-19 pandemic, households use various coping strategies to maintain an appropriate level of consumption. In the present study, it can be seen that 77% of households with an economic shock (losing part of income or illness of working member or other members) used at least one of the different strategies such as taking a loan, borrowing money, spending from savings, selling their properties in addition to using their current incomes. Figure 2 shows that the most frequently used strategy in this situation can be taking a loan and reducing expenditure, in other words cutting down on health expenditure or choosing cheaper services. Reducing the amount or the quality of food and diet or skipping meals can lead to deeper poverty. The percentage of households that use the strategy of selling assets or properties is significantly lower than the other strategies used. This could be the result of the high rate of poverty in Afghanistan.

## Discussion

This is the first study on the catastrophic and impoverishing effects of OOP health payments in Afghanistan, which generated indicators of financial protection and provided a better assessment of the health financing progress towards UHC. It helps to monitor and highlight health financing and coverage with an insight for the COVID-19 outbreak that has induced more vulnerable households and showed that health financing and coverage are ineffective in Afghanistan. The indicators derived from Afghanistan raise health equity concerns and how to mitigate inequities in health financing. Our study highlights the effects of OOP health payments when exposed to CHE and impoverishment and helps to explore the impacts of economic and health shocks related to the consequences of the COVID-19 outbreak. The decline of economic growth and loss of jobs due to the pandemic has significantly contributed to the increased vulnerability of households, which may push them to face further financial hardship. The financial protection indicators generated in this study provide evidence on health financing issues and guidance for feasible progress towards UHC in a normal situation or crisis [19].

Evidence reveals that the high contribution of OOPs for health services results in inappropriate coverage of social protection of health and economic hardships especially in Afghanistan as in developing countries [20]. These countries still suffer from the barriers to providing health financing resources, and ultimately, a high level of OOP payments should be paid, and it would lead to the imposition of CHE [21].

In Afghanistan, the share of Current Health Expenditure from GDP increased from 10% to 12.7% from 2008 to 2017. However, General Government Health Expenditure as a percentage of GDP decreased from 0.56% in 2008 to 0.49% in 2017 [8] that indicated a low level of the fiscal capacity of the government and its poor commitment to health compared to other uses of public spending [22]. General Government Health Expenditure as a percentage of General Government Expenditure is an indicator of the priority that a government gives to funding health relative to other public expenditure and in Afghanistan, it decreased from 3.4% in 2005 to 2.0% in 2016. This figure indicates that Afghan governments place less priority on health services. It can be because of political issues, conflict, and economic factors in Afghanistan. 

For many years, high OOP in Afghanistan, which was also confirmed by our study, has brought about an immense financial burden for many households. In the East Mediterranean Region (EMR) of the WHO, the OOP share in health expenditures varied from 0.1 to 84% with an average of 53% [9] compared to other countries such as Iran and Palestine (42%), Pakistan (60%), and Tunisia (38%) [22]. The WHO report indicates that 11.6% of the region population (76.9 million) are facing CHE (as 10% of total expenditure) [23]. The same report shows that in these countries, the total population pushed under the poverty line by OOPs was 1.26% based on the 3.20$ (PPP-2011) per day poverty line, and it was 0.39% based on 1.90$ a day in 2011 PPP [23]. Our findings showed that 32 and 14% of Afghan households are facing CHE in 2017, respectively to threshold 10% of total expenditure and 40% of non-food expenditure. This is considerably higher than the majority of EMR countries, 26.2% in Egypt (2012), 18.4% in Tunisia (2015), 15.8% in Yemen (2014), 15.8% in Iran (2013), and 4.5% in Pakistan (2015) using 10% threshold of total expenditure [24]. Results on the impoverishing effects indicated that 7% of the population in Afghanistan are pushed below the poverty rate (1.90$ a day in 2011 PPP), again, this is significantly higher than the majority of EMR countries, 3.5% in Yemen (2014), 0.12% Palestine (2016), 0.12% in Egyptian (2012), 0.09% in Tunisian (2015), 0.01% in Iran (2013), 0.87% in Pakistan (2015) [24].

Many countries are providing aids to protect vulnerable households during this COVID-19 crisis to reduce their exposure to CHE and impoverishment. A lesson learned from the COVID-19 crisis is that many countries were pushed to prioritize healthcare services to improve the information system for better targeting and to protect vulnerable groups. In Afghanistan, an essential benefit package should be prioritized and supported by domestic public financing. Furthermore, it should be cost-effective and through the capacity of the health delivery system to ensure equitable access.

In Afghanistan, there is a vital need to strengthen the financial health protection system. Our study emphasizes this necessity by not only providing indicators of financial health protection but also by offering an insight into the profile of households that are more exposed to CHE and impoverishment. The logistic regression results (Table 5) provide evidence of factors pushing versus protecting from financial hardship. In Afghanistan, it can be inferred that having an educated househould head or being employed are protective factors against CHE while the households having a female or senior as a family head, or having an elderly, sick, or disabled member are the risk factors of CHE. Furthermore, the ones living in rural and nomadic areas, or the households facing an economic shock are more likely to experience CHE and impoverishment due to direct payments on health. Understanding the distribution of the burden across sub-population groups will be particularly important as countries implement changes to their health financing policies.

Our results show that the percentage of households exposed to CHE in Afghanistan is significantly higher in families with a sick member. Households with diseases are suffering from the high costs of treatment. Our results indicated that the rate of CHE was at the highest among households with at least a member with “circulatory system and endocrine disease”. Studies from other countries including Iran, India, Nepal, Tanzania, and Korea showed that the types of diseases that affect financial hardship of OOPs most are infectious diseases, cancers, and renal diseases respectively [25].. Therefore, more attention and should be given to the health protection of the aforementioned households and the implementation of benefit packages and health coverage. The government can initiate and finance a health program to cover households facing CHE with particular diseases. We emphasize the need to set a coherent basic package of services including these diseases without compromising the transition toward UHC.

In Afghanistan, as in many countries, the presence of an elderly or a disabled person in the household has been found as a significant factor associated with CHE. These households face higher health expenditure due to the likelihood of several illnesses and a greater and long-term need for health services, yet they lack financial resources. In the absence of effective protection mechanisms, these groups face continuous risks of both financial hardship and poor health. Therefore, a targeted program for individuals with special attention should be provided to protect these households against CHE. This plan could be through financial exemptions such as covering services for free or may include minimal user fee charges to deter over-utilization of services, coverage of most commonly used cost-effective medicines in benefit packages, or providing different sets of social health protection, long-term care, and disability, so an integrated, poverty-oriented social policy approach is needed to address the particular needs of older persons [26, 27],

Our results showed a significantly higher risk of CHE for female-headed households in line with other studies [28]. In many countries in EMR, gender equity is a challenging issue, and especially in Afghanistan women are at higher risk of forgoing health care. Results from a study showed in Afghanistan among women who had delivered none of their children in a health center, money to pay for services appeared to be the most important barrier to accessing institutional delivery [29]. Financial barriers, family restrictions, and also cultural constraints make inequalities and disparities in accessing health care in Afghanistan, which requires special support for households with female householders. As a labor market aspect, results for 2019 showed labor force participation rate is 75% among Afghan males and 22% among females [30].

Similar to previous studies [17, 31–33], education and employment are protective factors against exposure to CHE. Households headed by a person with a high level of education have a low incidence of CHE in Afghanistan, which confirms the evidence that a high level of education increases the likelihood to have a better attitude towards health, to be less exposed to serious health conditions, and to have regular income to pay for health services in due time [17]..

Our results confirm that households with unemployed heads are more likely to experience CHE. COVID-19 could exacerbate the situation as many individuals became unemployed because of closures during the pandemic, especially if health problems precipitated the job loss. Consistent with our findings, a study using Korea Health Panel Survey data from 2014 to 2015 also showed those who quit economic activities were more prone to CHE than those who continued to engage in economic activities [34]. Therefore, efforts are needed to expand coverage for those people who suffer from high medical expenses. We know that the COVID-19 shocks are expected to increase unaffordability to healthcare services and expose further households facing financial hardship. If people face concerns about health care affordability, they may even delay seeking treatment or be prevented from obtaining the services they need. This makes the outbreak hard to control and puts the lives of many at risk. The further financial burden of COVID-19 is a huge challenge that cannot be tackled by using the same old tactics. It is timely and vital to have a healthy exchange of knowledge and learn to combine international recommendations with local creativity. In this situation, governments should increase social protection measures for individuals and employers for the workdays lost due to quarantine requirements as compensations, whether financial or in-kind such as paid sick leaves or wage subsidies, unemployment insurance with relaxed eligibility for those laid off, extended duration, or increased benefits, subsidies to firms affected by shutdowns or low demand, tax reliefs, or tax incentives for firms and households in the most vulnerable sectors [35, 36].

Similar to other studies [37, 38], our finding revealed that Afghan households in rural and nomadic areas faced a high risk of CHE because of their need to consult initially at a health center or district hospital. Some studies from other countries showed the same results and confirmed the lack of health facilities in villages, the cost and time needed to access urban services, and the indirect costs of accommodation in the urban area, and the inability of those in rural areas to cover these costs [17, 32]. Households headed by those with lower levels of education are often in rural areas, with inadequate access to health services. Governments’ responsibilities to ensure that poor and vulnerable groups receive quality health services through a well-functioning primary health care network should not be undermined. For low-income countries, a free, basic, primary health care package for all, financed by government tax revenue and donors is the only viable alternative [26].

Consistent with other studies from Iran [39], Turkey [40], and Thailand [41], our findings showed that better-off households are also at the risk of CHE because they seek and can spend more money on health services and the highest proportion of CHE is not always experienced by the lowest income group. The first reason for this pattern can be the prevalence of unmet needs in Afghanistan. Although measuring unmet needs through ALCS is not possible, but based on our results (Table 1) we know that just 47% of the poorest households and 67% of the richest households sought health care. It may be because some poor households delay seeking medical treatment as they cannot afford health care expenditures and, thus, are not regarded as incurring CHE as their health expenditure is zero. A study in 2005 and 2013 in Afghanistan showed a striking increase in the proportion for whom health care was not available (31 to 56%) and whose experience of coverage of health-care needs was negative (23 to 55%) [42]. The results of this study are consistent in terms of methodological issues and findings with other studies like [43], which state that in some low-income level countries respondents reported not seeking health care due to financial barriers (37% in Yemen, 20% for Lebanon). The other reason can be that most poor people have lower physical access to health care services. Consequently, more targeted measures are of critical importance to identify and protect the households who cannot afford and forgo health care services or cannot access them easily. Regular monitoring of prevalence of unmet need, household surveys for different groups, and also offering services including outpatient, inpatient, dentistry, and medicines can provide a clear picture of the availability of health services and the comprehensive benefits package for policymakers. Another reason for the higher rate of CHE among middle and rich classes in Afghanistan might be the ineffective health coverage, the recourse to private and expensive facilities to seek better quality health services as well as auto-medication.

Health care access barriers play a vital role in the health inequalities and disparities that exist in a population. The availability of health care services does not guarantee that they will be used by patients. The non-financial burden of health care seeking in Afghanistan falls into several issues. Firstly, in addition to the lack of availability of services and affordability of health care [44], 86.7% of the population can access health services within 2 hours, using any means of transportation. Results of a survey study in 2014 indicated rural populations also tend to pay around nine times more than the urban population for a one-way trip to a health facility. These showed that Afghanistan is still struggling to extend access to basic health services [5]. Secondly, due to social norms, health-seeking patterns are often different for men and women. Awareness of gender-influenced health-seeking patterns is a key component of gender-responsive health service delivery and essential to enhance timely and appropriate use of health services by both women and men to decrease gender-related access barriers to health care services in Afghanistan [44]. Higgins-Steele and Burke et all concluded that family restrictions or cultural constraints were the most important barrier to institutional delivery after financial reasons and limited transportation available [29]. Thirdly, for vulnerable groups, the perceived availability of health care and experience with coverage has not greatly improved over the last 10 years. Some segments of the population in Afghanistan, such as migrants, refugees, or other non-residents, may be at higher risk of not being able to access health care services or are accessing only a limited range of services. Therefore, access should be maintained and monitored regularly.

Afghanistan’s economy has been hard-hit by the outbreak of the COVID-19 virus, due to negative impacts on consumption, exports, and remittances. The scale of the estimated decline in GDP growth is up to 5.5% by WB forecast and 3.0% by International Monetary Fund forecast for 2020 in Afghanistan. On the other hand, the displacement crisis persists, driven by intensified government and Taliban operations in the context of political negotiations. People in Afghanistan before COVID-19 were already at high risk of poverty and the outbreak can push the population further under poverty.

In order to protect households from financial hardship, especially during the COVID-19 pandemic, government policies should meet the healthcare needs of those with increased health needs as well as the ones of lower socioeconomic status. The most recommended policy by international experts is to remove all financial barriers to access to health services. To protect households, eliminating user fees for COVID-19 health services and suspending fees for all other essential health services, at least during the pandemic, are crucial. This can protect people from financial hardship but does not guarantee that all will have access to the needed essential health care services. Some households are at higher risk of not being able to access health care services or they can only access a limited range of services. Effective responses should include extending entitlement to particularly publicly financed health services to all, regardless of residence as well [35, 45]. Protection of households by removing the direct financial barriers to access is not enough since there are other indirect financial barriers such as transportation costs, long waiting lists, distance to healthcare, required travel time, and informal charges. During the pandemic, households are at risk of losing their incomes due to job loss, part of their wages, and consequently leading to an increase in poverty. Therefore, the government should expand coverage of income supports, increase social benefits, and make these more accessible by simplifying and accelerating administrative processes to prevent an increase in poverty.

Our results showed households that faced CHE used different strategies to pay for health care services: borrowing or taking a loan (39%), using savings (23%), using regular salary (19%), and selling house, land, or assets (10%). The results of this study confirm that developing and improving an effective financial health protection strategy is crucial to establishing a more equitable and sustainable health financing system as a long-term goal for Afghanistan. Estimating the needs and current gap in financial resources required for an effective response phase and developing a contingency financing plan to ensure sufficient and identifying cost-effective options for implementing the response strategy and establishing the basis for a longer-term recovery plan could be considerable [35].

In Afghanistan, health financing has several major problems that cause high OOPs and hence financial hardship in access to basic health services for households. It is obvious that government investment in health is very low compared to neighboring or regional countries and urgent actions are needed through increasing domestic government health spending, reducing OOP health spending, and eliminating inefficiencies in healthcare. In an attempt to raise revenues, MoPH/HEFD has developed several policy papers and strategic frameworks: “Health Financing Policy 2012-2020” focused on the generation of domestic resources for health through taxation and prepayment mechanisms; Policy brief statement 2016–4 “Revenue collection and management - A Challenge to the Afghan Government” outlined revenue collection challenges and proposed various policy options. Health Financing Strategy 2019–2023 developed in 2019 outlined Strategic Directions and Objectives and promoted efficiency gains in each Strategic Objective including performance monitoring framework with baseline and targets.

Although there were no formal user fees in public health facilities in Afghanistan before COVID-19 [46], there are many concerns related to high OOP spending by households [47] and also the quality and utilization of primary healthcare services after removing user fees in public health services [48]. During the COVID-19 pandemic, Afghanistan COVID-19 Emergency Response and Health Systems Preparedness Project aimed to respond and mitigate the threat posed by COVID-19 in Afghanistan by strengthening national systems for public health preparedness and essential health care service delivery help to provide the best care possible for people who become ill despite a surge in demand. It will also ensure ongoing support for people ill in the community to minimize the overall impact of the disease on society, public services and on the economy [49].

Despite the efforts, the existing revenue collection model is still fragmented and does not allow pooling funds at a regional or national level. There is still no strong mechanism in place to pool public, private, and external resources and thus to improve financial risk protection against catastrophic health expenditures and impoverishment. High informal payments deter individuals to seek needed healthcare services. Substantial efforts are needed to optimize the basic package of services and ensure universal access to basic services. Lack of strategic purchasing and inefficient provider payment mechanisms in public and NGOs are constituting further problems to the already fragile health financing system in Afghanistan. Further, there is poor public finance management at the national and sub-national levels. Better leadership and more effective management of public financing are the keys to support health financing and purchasing reforms.

Afghanistan health care still suffers from ineffective leadership and governance, centralized decision-making, and low participation of key actors in health-related decision making. Furthermore, limited use of evidence and poor recognition of contextual influences lead to ineffective use of resources and poor decision making. The health management information system contains aggregated information at the level of districts and healthcare institutions; however, it does not contain personalized patient data, which limits its use. The current lack of data needs to be addressed and health information systems should be improved so as to create a database of recent and relevant data, which will allow a better response to current and future health issues. More importantly, indicators should be developed for all the above-mentioned issues, so their progress can be tracked.

Decades of war and civil strife have adversely affected the coverage and the delivery of health services in Afghanistan. Afghanistan still suffers from fragile socio-economic status and political instability as well as low literacy, insufficient infrastructure, weak public sector policies, and significant dependence on foreign aid. In this context, the government has initiated transformation health reforms through building institutional, governance, and human resource capacity. The local government that still needs support, is implicated in follow-up and monitor the feasibilities of these reforms. To be effective, support to local government requires a strong national sector strategy that: recognizes diverse capacity needs, includes a dedicated budget for institutional support, and provides for capacity building and training. Building the local institutional capacity is essential for better targeting vulnerable groups’ ongoing financial risk protection.

We faced some challenges in analyzing the ALCS data. As a first limitation, due to the nature of the statistics scheme, we lacked access to some variables recommended in other studies. We believe there are other influential variables information about unmet needs and potential reasons that could be useful for policymakers to analyze the financial burden on households. In terms of recall periods, the results indicated a preference for longer recall periods in hospital spending and for shorter recall periods in outpatient and medication spending (50% of the surveys used these recall periods). Some validation studies showed that the probability of misreporting (possibly due to forgetting) increases when the gap between the time of the interview and the event increases [50]. The expenditure items are not asked according to Classification of Individual Consumption by Purpose (COICOP) and it seems we can estimate total consumption accurately enough. In general, more detailed items yield higher aggregate expenditure estimates but there is no conclusion on the optimal number of breakdown items. The number of breakdown items is usually decided based on the purpose of the survey. For example, a health-focused survey may contain more than 10 items of health expenditure if the purpose is to know how much households pay for different services in different facilities; while in a general-purpose survey, health expenditure may only be presented with one or two questions.

## Conclusion

The findings showed severe CHE incidence and impoverishment in Afghanistan. Households in Afghanistan are dealing with a financial burden due to high OOPs and poverty, generating that 32% of them are facing CHE in 2017 which is among the highest rate in the world. Logistic regression showed that an economic shock, having an unemployed head, a female head, an elderly member, a disabled or sick member, or living in rural or nomadic areas increase catastrophic health spending in Afghanistan and hence increase impoverished population.

With these evidences of the high burden of financial risk protection, Afghanistan is still facing multiple socio-economic challenges, an ongoing critical juncture of the peace process, and severely hit by the COVID-19 pandemic. The health and economic shocks of COVID-19 will certainly exacerbate in the near future, by increasing vulnerability and financial hardship of the Afghani population. This study perhaps the earliest to comprehensively evaluate the impacts of COVID-19 but it describes the most affected households by the CHE and impoverishment and provides great support to prevent these risks.

In view to achieve UHC-SDG, public health services need to be decentralized and equitably distributed, and to be provided affordably and in sufficient quality. Furthermore, the results of this study call for the need for a strategy of health financing for strengthening and addressing social health protection to achieve UHC. Precisely, the results of this study recommend that the government of Afghanistan should emphasize in its financing strategy to think of increasing public expenditure and reduce OOP expenditure on health, developing a social health coverage scheme, and define a broader and affordable priority benefit package. A specific objective of the strategy could be targeting subsidies and financial protection for households incurring CHE.

Findings from this study would be supportive for monitoring the progression towards UHC but other studies exploring how to reduce OOP and the impact of OOP payment for specific diseases on catastrophic expenditure are recommended.

## Data Availability

The datasets analyzed during the current study are not publicly available due to National Statistic and Information Authority rules for publishing but are available from the corresponding author on reasonable request.

## Abbreviations

FP: Financial Protection
OOPs: Out of Pocket spending
UHC: Universal Health Coverage
SDG: Sustainable Development Goals
CHE: Catastrophic Health Expenditure
BPHS: Basic Package of Health Services
EPHS: Essential Package of Hospital Services
WB: World Bank
GDP: Gross Domestic Product
EMR: East Mediterranean Region
